# What’s the use?

**DOI:** 10.7554/eLife.73193

**Published:** 2021-10-12

**Authors:** Jason M Fletcher, Yuchang Wu, Qiongshi Lu

**Affiliations:** 1 Department of Sociology, University of Wisconsin-Madison Madison United States; 2 La Follette School of Public Affairs, University of Wisconsin-Madison Madison United States; 3 Center for Demography of Health and Aging, University of Wisconsin-Madison Madison United States; 4 Department of Biostatistics and Medical Informatics, University of Wisconsin-Madison Madison United States

**Keywords:** polygenic risk scores, embryo, preimplantation genetic testing, in vitro fertilization, Human

## Abstract

A theoretical framework predicts that using polygenic screening to select embryos against traits that depend on many genes has few benefits.

**Related research article** Lencz T, Backenroth D, Granot-Hershkovitz E, Green A, Gettler K, Cho J, Weissbrod O, Zuk O, Carmi S. 2021. Utility of polygenic embryo screening for disease depends on the selection strategy. *eLife* **10**:e64716. doi: 10.7554/eLife.64716

Using genetic tests to select ‘designer babies’ has been a subject of science fiction for generations, but it is now getting closer to becoming a reality. Like in many other areas of science, techniques are progressing much faster than ethical and policy discussions (Conley and Fletcher, 2017). Indeed, many parents already genetically screen embryos produced using in vitro fertilization (IVF) to avoid passing on genetic variants that are known to directly cause genetic disorders (Baruch et al., 2008).

However, the plummeting cost of genetic sequencing and the sophistication of the tools used to predict characteristics based on the genetics of an individual will soon make it possible to screen for ‘complex traits’ – this is, traits that depend on many genes. This type of analysis is called polygenic screening. In short, it assesses the probability of an embryo exhibiting a trait (such as a health condition) based on the collection of genetic variants it carries that are known to influence that trait. Polygenic screening could allow couples to produce several embryos through IVF, check how likely each one is to manifest one or several complex traits, and, based on that information, decide which embryo to implant. Unlike screening for conditions that depend on a single gene, however, polygenic screening cannot always guarantee that an embryo will manifest a condition. The traits that can be tested for using polygenic screening include many health outcomes, but also characteristics that are more controversial to target, such as the IQ of a future baby (Lázaro-Muñoz et al., 2021).

Now, in eLife, Todd Lencz (Zucker School of Medicine at Hofstra/Northwell and Northwell Health), Shai Carmi (Hebrew University of Jerusalem) and colleagues – including Daniel Backenroth (Hebrew University) as joint first author with Lencz – report on the usefulness of polygenic screening when testing for complex health traits, such as schizophrenia and Crohn’s disease (Lencz et al., 2021). Briefly, the utility of screening will depend on the predictive accuracy of the genetic tests performed and, more subtly, on the goal of the testing as well as the setting – that is, who specifically is being tested, and for what.

Lencz et al. largely take predictive accuracy as a given (but see Fletcher et al., 2021 for issues of accuracy), and employ a theoretical framework to ask deeper questions about the usefulness of several strategies that can be used when selecting embryos. First, Lencz et al. assume that a hypothetical couple produces several viable embryos via IVF. The probability of each of those embryos having a specific complex trait (for example, a given health condition) can be determined using polygenic screening. Based on these probabilities, the couple must then choose which embryo to implant. Starting from these assumptions, Lencz et al. compare the utility of different approaches to selection. Finally, Lencz et al. use genome data from schizophrenia and Crohn’s disease case-control studies to simulate virtual couples and their offspring and confirm their predictions.

Lencz et al. found that, in general, polygenic screening is not very useful when it targets complex health traits. This is because most selections occur between embryos with the same parents, which substantially limits both genetic and environmental variability. Thus, when selecting for specific characteristics, there is only a small number of possible outcomes, which reduces the usefulness of any selection regime. But, within this constraint, focusing on binary traits (for example, an embryo having or not having a disease) illuminates an important asymmetry in the utility of selection. Take, for example, the case of a couple having to select one of five embryos for implantation. The benefits of ruling out the one or two embryos with the highest risk scores, and then selecting from the remaining three or four embryos by chance are small. This is because although a couple is choosing at random between three embryos that do not have the highest risk of the disease, each of them could still be at a moderate risk of having the disease. Instead, a better strategy is to pick the embryo with the lowest risk score.

However, this result provides the first reason why parents, if they are well-informed, are unlikely to perform polygenic screening. Assuming that the negative attitudes most Americans have against enhancing traits through gene editing extend to genetic screening, parents are likely to want to screen embryos to avoid disease, but are probably against choosing the ‘best’ embryo (Scheufele et al., 2017).

Even if parents (counter to intuition) did want to choose the ‘best’ embryo, deciding which is the ‘best’ soon becomes an impossible task. Lencz et al. only consider the situation where parents are choosing between embryos more or less likely to exhibit one or two complex traits; but what happens when several traits, each dependent on many genes, are of interest? A decrease in the risk of one health condition, for example, could lead to an increase in the risk of another. Trading off risks between schizophrenia and Crohn’s disease would be difficult, but when other characteristics such as genetic risks for height, IQ, and eye color are thrown into the mix, the decision becomes impossible. This ‘paradox of choice’ is a second reason that parents will not choose polygenic screening if they are properly informed about it (Schwartz, 2004).

For both of these reasons, the analysis by Lencz et al. places critical focus on the lack of utility of genetic screening for complex traits, and the findings are an incredibly important contribution for science and for public and policy discussions. Nevertheless, a number of questions about the value of polygenic screening still remain. Lencz et al. answer the (constrained) question of which embryo to choose if you must choose one. Indeed, if there are five embryos with similar risks to pick from, Lencz et al. assume that parents will select one randomly. A related question is whether these parents should implant one of the five embryos they already have, or wait to use others. This may be answered using a method that predicts whether a couple are likely to produce a lower-risk embryo than the ones they already have, based on the parents’ genetic information (Chen et al., 2020).

An obvious next step will be to study actual couples (and not virtual couples as done by Lencz et al.) because couples in the real world are likely to be more genetically similar than couples chosen at random (Domingue et al., 2014; Conley et al., 2016). Like the selection question raised by Lencz et al., this use of polygenic screening opens a large set of ethical questions as well as questions about the utility of the approach that are not yet fully answered.
